# What information did the DSM-5 Cultural Formulation Interviews provide when used with Swedish-speaking patients in a psychiatric setting in Stockholm?

**DOI:** 10.3389/fpsyt.2024.1377006

**Published:** 2024-05-16

**Authors:** Malin Idar Wallin, Valerie DeMarinis, Lauri Nevonen, Sofie Bäärnhielm

**Affiliations:** ^1^Center for Psychiatry Research, Department of Clinical Neuroscience, Karolinska Institutet (KI) & Stockholm Health Care Services, Stockholm, Sweden; ^2^Transcultural Centre, Region Stockholm, Stockholm, Sweden; ^3^Department of Public Health and Clinical Medicine, Faculty of Medicine, Umeå University, Umeå, Västerbotten, Sweden; ^4^Division Mental Health Care, Innlandet Hospital Trust, Hamar, Norway; ^5^Department of Medical Sciences, Faculty of Medicine and Health, Örebro University, Örebro, Sweden; ^6^Aleris Psychiatry Täby, Stockholm, Sweden

**Keywords:** cultural formulation, cultural psychiatry, clinical assessment, ethnicity and mental health, cultural identity

## Abstract

**Introduction:**

Cultural and contextual factors affect communication and how psychiatric symptoms are presented, therefore psychiatric assessments need to include awareness of the patients’ culture and context. The Cultural Formulation Interview (CFI) in DSM-5 is a person-centred tool developed to support the exploration of cultural and contextual factors in an individualized and non-stereotypic way.

**Methods:**

The aim of this qualitative study was to find out what information the DSM-5 CFI revealed when used with native Swedish-speaking patients as part of routine clinical psychiatric assessment at an outpatient clinic. An additional aim was to enhance understanding of what kind of information the questions about background and identity yielded. The CFI was added to the psychiatric assessment of 62 native Swedish-speaking patients at an outpatient psychiatric clinic in Stockholm.

**Results:**

From the thematic analysis of the documented CFI answers, six central themes were found; *Descriptions of distress and dysfunction, Managing problems and distress, Current life conditions affecting the person, Perceived failure in meeting social expectations, Making sense of the problem, and Experiences of, and wishes for, help.* The CFI questions about identity yielded much information, mainly related to social position and feelings of social failure.

**Discussion:**

For further refinement of the CFI, we see a need for re-framing the questions about cultural identity and its impact on health so that they are better understood. This is needed for majority population patients as direct questions about culture may be difficult to understand when cultural norms are implicit and often unexamined. For clinical implications, our findings suggest that for cultural majority patients the DSM-5 CFI can be a useful person-centred tool for exploring cultural and, in particular, social factors and patients’ perception and understanding of distress.

## Introduction

Psychiatric assessments are a complex process that includes reaching an understanding of the patient’s problems, identifying psychiatric disorders, and evaluating treatment options, (1, 2). Cultural and contextual factors affect communication and how psychiatric symptoms are presented. The impact of the patients’ culture and context need to be explored in an individualized way to be able to provide person-centred care (1, 2), which refers to patient’s desire for information and for sharing decision making to be considered and responded to appropriately (3).

The person-centred DSM-5 CFI aims to facilitate communication and the exploration of cultural and contextual factors of importance in the clinical encounter, such as cultural definitions of the problem and causes, as well as stressors, support and the role of cultural identity. Evaluations of the CFI show that its use can improve patient – clinician alliance and communication (4, 5), as well as contribute with contextual information and support trust building and treatment planning (6). The CFI is intended to be used in the initial psychiatric assessment with all patients, although it could be especially helpful when the patient and the clinician do not share the same social or cultural background (1, 2). The CFI can also be used to understand cultural and social barriers better, such as misunderstandings or differences in expectations, between patient and clinician as well as for assessing illness severity. The CFI includes 16 questions related to four domains: 1) definition of the problem, 2) perception of cause, context and support, including the role of cultural identity 3) self-coping and past help seeking, and 4) current help seeking. The open questions of the CFI provide space for patients to elaborate and describe their perceived problems and distress (7) and are intended to increase commitment and adherence to treatment (1, 2). The CFI is meant to be an additional instrument for identifying relevant cultural and contextual information and does not replace traditional diagnostic tools and skills (8).

The CFI is being implemented and used internationally and has been evaluated in diverse cultural contexts (9–16). In an international field trial, a slightly different precursor of the CFI was found to be a feasible, acceptable and clinically useful tool (17), and the positive results correspond with later research (6, 7, 12). In an RCT, the CFI effect on psychiatric diagnostics was evaluated and found to contribute to identifying depression disorder diagnoses, as well as facilitate differential diagnostics, among non-native speaking patients referred to a Swedish psychiatric outpatient clinic (15). A thematic analysis of documented information from CFI with non-native Swedish-speaking patients, that participated in the aforementioned RCT, showed that the CFI stimulated patients’ sharing of information related to problem description and problem management as well as their present life situation. The CFI information contained contextualized descriptions of dysfunction and current life conditions, as well as expressions of emotions, often described along with somatic terms (18).

Although the CFI seems to facilitate communication and alliance building in healthcare encounters (4, 5), there are data pointing to needs for improvement and refinement. It has been criticized for not including questions related to social determinants of health, like social structures of, e.g. employment or educational opportunities, housing or racial and ethnic disparities (11, 19). Several studies have empathized the complexity of the identity concept, and the CFI questions about background and identity have been found to be abstract and difficult for some patients to understand (6, 7, 14, 20, 21). Furthermore, the CFI has commonly been used to understand cultural barriers between patient and clinician, and most previous studies have evaluated the CFI when used with minority populations. To the best of our knowledge, there is a deficiency of studies analysing what information the CFI provides when used with the majority population in routine psychiatric assessments. As the CFI is intended to be used with all patients, for further implementation and improvement, there is a need to understand what information the CFI stimulates to be shared in psychiatric assessments with patients from majority populations.

The overall aim of this study is to find out what information the CFI questions revealed when used with native Swedish-speaking patients, as part of routine clinical psychiatric assessment at an outpatient clinic. An additional aim is to enhance understanding of what kind of information the questions about background and identity yielded.

## Materials and methods

In this study, documented CFI answers from native Swedish-speaking patients were analysed. The CFI answers originate from the aforementioned randomized control trial (RCT) evaluating the CFI effect on psychiatric diagnostics. In the RCT, the CFI was used with an intervention group in the diagnostic process of new patients, in addition to ordinary diagnostic procedure.

### Setting

The setting, from which the data originate, includes three outpatient care clinics in the multicultural Järva area in the western suburbs of Stockholm, Sweden. The social situation in most parts of the area is strained, with unemployment and low-income approximately twice as common as in the Stockholm region in general (22). Some parts of the area evidence higher economic incomes with a Swedish born population in the majority.

### Study procedure

The patients included in the RCT, conducted between August 2015 and May 2017, were new patients who had not been in contact with psychiatric care over the past two years. In the psychiatric assessments of the patients who were randomized to intervention, using a lottery system with equal likelihood of assignment, the CFI was used in addition to the standard psychiatric diagnostic procedure that was consistent for intervention and control patients. A total of 15 clinicians conducted the CFI during the study. At the start of the study, these clinicians participated in a two half-days training course, which included discussions based on their own clinical cases, as well as lectures and role play using the CFI. The professions of the clinicians were: 10 psychologists/psychotherapists, 3 psychiatric nurses, 1 counsellor, and 1 mental health auxiliary. The patients participating in the study were given oral and written information at the beginning of the consultation, including information about the participation being voluntary and that consent could be withdrawn at any time, without any negative consequences. All 16 questions of the core CFI were asked as early as possible in the consultation. However, this was not always during the first encounter and sometimes the interview was divided into two consultations due to lack of time. Due to earlier research noting concerns among new patients about having their consultations recorded, the clinicians documented the CFI answers by taking notes, later integrated in the patient’s electronic health record. The CFI answers were documented in a narrative way, question by question, often as detailed answers but sometimes more as a resumé related to each question. At the beginning of the study, the CFI questions were not always documented in order as questions 6-10 often were saved for later and documented just before question 16. When the clinicians were more used to the CFI and the study had been in progress for a while, the questions were asked in the intended order. To ask the questions in a different order was a choice made by the clinicians in the clinical situation. The documentation was sometimes written in a first person form, as the patient’s direct narrative with the pronoun, “I “. This contrasted with the rest of the text in the medical records where patient information was written in a third person form using he, she, or the patient pronouns.

Ethical approval for the research project was obtained from the Regional Ethical Review Board in Stockholm (2015/243-31/2).

### Sample and data

The sample of native Swedish-speaking patients was defined based on the information in their electronic health records as raised in Sweden and/or in Swedish-speaking families. Native Swedish-speaking patients can be seen as a proxy for cultural majority patients, in this study referring to not being a migrant. However, we do not know if these patients belong to an ethnic minority, or have another ethnicity (for example from one parent) other than Swedish.

#### Sample characteristics

Most patients were women (71%) and more than 35% of the patients were below 25 years old. 29% of the patients were unemployed (including students) and the most common completed educational level was college education (**Table 1**).

**Table 1:** Sociodemographic characteristics of the sample (N=62).

	N62 (%)
Sex:	
Women	44(71.0)
Men	18(29.0)
Missing	0(0)
Age group:	
17-24	22(35.5)
25-34	19(30.7)
35-44	10(16.1)
45-64	11(17.7)
Missing	0(0)
Employment group:	
Employed	31(50.0)
Not employed	18(29.0)
Welfare benefits	12(19.4)
Missing	1(1.6)
Educational level:	
Elementary school or lower	14(22.6)
College	26(42.0)
Senior high school	11(17.7)
Missing	11(17.7)
Country of origin:^^1^^	
Sweden	62(100)
Missing	0(0.0)
Native language:	
Swedish	62(100)
Missing	0(0.0)

The data chosen for the qualitative analysis comprise the full data set of the 62 CFI answers from the native Swedish-speaking patients in the RCT, documented in the patients’ medical health records by the clinicians conducting the CFI. The patients were referred to the clinic along with information, sometimes containing a suspicion of a diagnosis, mostly depression (30%), anxiety (20%) or suspicions of a neuropsychiatric disorder (22%).

### Analysis

The CFI answers documented in the patients’ medical health records were copied into one shared document and analysed through qualitative thematic analysis (23). We chose to make the analysis of the whole material and not question by question as the patients did not always answer the question being asked but another one. The analysis was made in six steps. First, we identified meaning making units in 10 of the documented CFIs. Second, to reach a structure for further coding of the material, conversational topics (categories) and a preliminary coding scheme were constructed from the meaning making units to facilitate further analysis of the whole data set. Third, the coding scheme was continually developed and revised during the coding of new interviews. Fourth, the categories were grouped into sub-themes. Fifth, the sub-themes were grouped into themes (23). Sixth, to minimize the risk of fragmentation and misinterpretations of the text the meaning units under each overarching theme were analysed once more and the material in its entirety was carefully reviewed.

A multi-coder process was used when conducting the analysis. Preliminary codes, categories, sub-themes and themes were developed by the first author. In association with the last author, the codes, categories, and later the sub-themes and themes were discussed and revised, until an agreement was reached about the interpretation of the content. The analysis and interpretation of the material were discussed with the second author, and in the next step all the authors were involved in revisions of the themes and sub-themes. The software program Nvivo12 (24) was used to support the analysis process.

## Results

The analysis, including all information from the 62 CFI answers documented in the patients’ health records, reveals information written in a narrative form. From the qualitative thematic analysis, the following six themes were found;

Descriptions of distress and dysfunction, Managing problems and distress, Current life conditions affecting the person, Perceived failure in meeting social expectations, Making sense of the problem, and Experiences of, and wishes for, help (**Table 2**).

**Table 2:** Presentation of themes and sub-themes with examples of related meaning units.

Descriptions of distress and dysfunction 401*	*Limiting consequences in everyday life*	*“I am actually a social person. Now I don’t take any initiatives to make contact.”*
	*Emotions and behaviours related to dysfunction*	*“The worst thing is the worry about how things will turn out in a larger and longer perspective.”*
	*Psychiatric wordings of distress*	*“I get depressed on and off. I have problems with anxiety.”*
	*The personal history of the problem*	*“That I’m depressed, that I’ve had problems with it for several years.”*
Managing problems and distress 186*	*Strategies for managing the problem*	*“Listening to music can be an alternative to cope when I feel bad.”*
	*Current self-positioning to the problem*	*“For a long time I refused to accept that I feel mentally ill … I thought it would pass.”*
	*Deciding if and how to share*	*“Don’t want to burden them, they have their own lives.”*
Current life conditions affecting the person 249*	*Social stress factors*	*“Am stressed by the social security authorities. Feels like a threat.”*
	*The impact of others on the perceived problems*	*“My friend often says that you have to fight in order to survive.”*
Perceived failure in meeting failure in social expectations 159*	*Recognizing oneself in relation to values and roles*	*“The most important part of my identity is my profession.”*
	*Self-worth valued by performance*	*“Had many advantages growing up. I shouldn’t have failed.”*
	*Social norms and values of the family of origin*	*“Many in the family are successful.”*
	*Future social objectives*	*“Wants to gain energy and strength to tackle his studies. He wants to study at university in the future.”*
Making sense of the problem 188*	*Family- and childhood-related conditions*	*“Growing up with a substance abusing father, which led to an insecurity … It’s part of me, my history.”*
	*External stressful conditions*	*“The slump that happened this summer, which led to her seeking help now, was caused by stress and pressure at work.”*
	*Heredity and biology*	*“The cause is biological. Hereditary perhaps.”*
	*Psychological vulnerability*	*“She thinks her problems are stress-related. An internal rather than external stress.”*
	*Suspicion of a psychiatric diagnosis*	*“I have suspected it to be ADHD or bipolar disorder or both.”*
Experiences of, and wishes for, help 313*	*Help-seeking experiences*	*“Have had quite a few consultations. It has felt good, but basically not helped. The anxiety and depression remain.”*
	*Non-medical help seeking*	*“Tried mindfulness and acupuncture before which helped a lot.”*
	*Barriers to help seeking*	*“She wanted to manage it herself without help.”*
	*Ideas and thoughts about improvement*	*“Some kind of diagnosis … If I get a diagnosis, I have something to relate to. That is the way I am.”*
	*Concerns related to healthcare interventions*	*“I’m afraid of not being taken seriously.”*

*Number of references.

The amount of information related to each theme is not equal, and the theme that is richest in references is presented first and thereafter the themes are presented mainly in descending order.

When the documentation was written in the first person, the pronoun “I “ is used in the quotes, and when the documentation was written in the third person this form was retained in the quotes. For reasons of confidentiality, only short quotes are presented when reporting the results.

### Descriptions of distress and dysfunction

This is the most comprehensive theme and includes the patients’ descriptions of problems and their consequences. The patients narrate the limiting consequences, for social relations and in everyday life, and use emotional words (like worry or anxiety) and recount behaviours related to dysfunction. The use of psychiatric terms is common when describing the perceived distress. The patients also portray the problems from a personal life historical perspective, how the problems evolved over time, or, for some, they have always been there. The underlying sub-themes are: Limiting consequences in everyday life, Emotions and behaviours related to dysfunction, Psychiatric wordings of distress, and The personal history of the problem.

#### Limiting consequences in everyday life

In this sub-theme, the patients narrate how their problems or distress prevent them from doing things in everyday life or make it difficult to cope with ordinary tasks and demands. The patients picture how their problems have a negative impact on social relations through descriptions of distancing oneself from others and of isolation.

“I have fewer social contacts because I feel bad, I isolate myself. I am vulnerable if something were to happen.”

The patients describe sleeping difficulties and problems with memory loss and concentration difficulties.

“ Difficulty sleeping regularly or sleeping too much. Difficulty remembering things.”

Some patients perceive the problems as obstacles to the development of life itself.

#### Emotions and behaviours related to dysfunction

This sub-theme contains descriptions of emotions related to the patient’s distress. Common emotions described are worry, anxiety and fear.

“I feel so unbelievably bad from having anxiety and therefore I become afraid of the anxiety itself.”

Other commonly described emotions are tiredness, mood swings and feelings of panic. The sub-theme also includes dysfunctional behaviours related to the perceived distress. These behaviours are actions of compulsion and phobia as well as self-injurious behaviours.

#### Psychiatric wordings of distress

The use of psychiatric words is common among the patients when describing their perceived distress. Psychiatric wordings are used as part of their everyday vocabulary and are used often when describing the problems to others.

“To family and friends I say I have bipolar disorder.”

#### The personal history of the problem

This sub-theme contains descriptions of how the patients’ problems have evolved over time. Some describe how they have had the problems on and off for a long time or for as long as they can remember.

“I have gone in and out of depression.”

Other patients describe their distress as new to them and unknown.

### Managing problems and distress

This theme contains descriptions of the patients’ strategies and self-positioning to their distress as well as narratives of their decisions to share their problems with others or not. The sub-themes of this theme are: Strategies for managing the problem, Current self-positioning to the problem and Deciding if and how to share.

#### Strategies for managing the problem

The patients describe concrete strategies for managing their perceived distress and activities that relieve it. These strategies are mainly concrete actions like exercise, keeping busy or creating routines helpful in everyday life. There are also examples of adjustments of their daily life and efforts of thinking differently. Some patients describe activities like writing, knitting, or meditation used as strategies to feel better.

“Methods that have been helpful in not getting too low or losing focus and control are knitting or crocheting and keeping a diary.”

Examples of taking one day at a time, self-medicating by using drugs or forcing oneself to do or not to do things are also found.

#### Current self-positioning to the problem

The patients describe how they position themselves to the perceived problems and distress. The self-positioning differs among the patients, but shame and avoidance are frequently described.

“I have been ashamed of having a mental illness and not being able to deal with it.”

Not knowing how to relate, or finding a way to relate to the perceived distress are described by the patients. This can include keeping up an appearance or creating a facade towards others. Denial as well as destructive behaviours are also represented.

#### Deciding if and how to share

Patients describe their decision to tell others about their problems or not, who they tell and how they talk about their distress.

“Describe the problems to my partner in the same way as to you now. To others I say I ‘crashed’.”

Common ways to talk about the perceived distress involve trying to explain as openly as possible. The expressions of not sharing their problems with others are as many as the expressions of sharing. Some patients describe obstacles to talk about their problems and a reoccurring example is not wanting to burden others.

“There is a limit to how much a person can share. She (the patient) doesn’t want to ruin anyone’s day.”

Shame is another example of a reason for not sharing.

### Current life conditions affecting the person

This theme contains social stress factors, often related to work or economy, and the impact of others on the perceived problems, including others’ explanations of the problems. The sub-themes included are: Social stress factors and The impact of others on the perceived problems.

#### Social stress factors

When answering the CFI questions, the patients share social stress factors affecting them and their perceived distress, which are often related to work or economic strains. The most frequently mentioned stress factor is living under strained economic conditions. Stressful working conditions, often an uncertain job situation or being without a job, also frequently occur.

“Economic difficulties. Have an hourly job, irregular income … These are a definite stress factor.”

Other social stress factors are related to the family situation or family members. Frequently described is living with sick family members or in other ways burdensome family conditions. Some stress factors are of relational character, often strained relations to family members.

“The family problems, that they deny both my problems and sexual orientation, are a constant stress and sadness.”

An uncertain housing situation and a perceived social pressure also occur.

#### The impact of others on the perceived problems

This sub-theme illuminates the impact of others on the perceived distress, such as others’ views of what is causing the problems as well as what would be helpful. Others’ views span from biochemical or psychiatric explanations to stressful living conditions, thinking too much, or just being lazy.

“That I am at home too much and think too much. That I should make more effort.”

Others’ views of what would be helpful also vary from pushing oneself to do things to ignoring the problem. Other suggestions are exercise or assessment and treatment. There are also descriptions of others’ approach to the problems and if the patients perceive support from them or not.

“I have support from my husband. He feels stable and supports me when I have anxiety and get thrown off track.”

### Perceived failure in meeting sociocultural expectations

This theme captures the patients’ reflections on identity, values, cultural norms in their family of origin and in their context. The patients respond to the questions about identity mainly in relation to sociocultural expectations and their own values, often based on a social position. Future social objectives of an ability to meet social expectations are also presented. The sub-themes found are: Recognizing oneself in relation to values and roles, Self-worth valued by performance, Social norms and values of the family of origin, and Future social objectives.

#### Recognizing oneself in relation to values and roles

In this sub-theme the patients reveal their perception of identity and feelings of belonging to a certain group. Identity is often discussed in relation to an ability to perform and is related to a social role.

“The most important part of my identity is my work.”

Some patients describe their identity as formed by their childhood cultural context, which is sometimes perceived as problematic. Feelings of not fitting in and of relating one’s own identity to other cultural references are also represented. Some patients define themselves by losses in life or by being a failure.

“Being a failure is part of my identity.”

Feelings of losing one’s identity, of not belonging or feeling lost are also described. Desires of a feeling of belonging somewhere or to be like “everyone else” are also expressed.

“Feelings of being lost, that I don’t feel at home anywhere else than in my professional role.”

#### Self-worth valued by performance

This sub-theme highlights how the patients value their self-worth in relation to their ability to perform in accordance with norms and values. Disappointments and feelings of failure frequently occur related to ones’ own, or others’, expectations of performance.

“Can feel depressed because she is disappointed in herself. Would love for things to be perfect, but they won’t even be good.”

Sometimes the patients perceive the problem itself as an obstacle to fulfilling their own expectations, and sometimes the failure of expectations to perform is perceived as causing the distress. Failure to perform is often described in relation to others’ potential disappointment, as well as ones’ own.

“He feels that he must constantly perform at his peak so that others (and himself) will not be disappointed.”

The patients express feelings of not living in accordance with one’s own values and expectations.

“That’s not the person I want to be.”

Some patients even describe this being the main actual problem. A conflict between one’s own identity, of visions of life and how life turned out with limitations of ones’ own capacity and abilities to achieve, is described.

“I often compare myself with who I was when I grew up and what I wanted then. It didn’t turn out that way. I feel lost that life didn’t turn out the way I thought it would.”

A perception of living conditions where one “should succeed” is described, and one patient expresses how living in a country and in a time where everything is possible and still failing might contribute to feelings of failure and distress.

“She believes that part of her problem is based on the fact that she comes from a generation and a country where there is every opportunity to succeed if you want to.”

#### Social norms and values of family of origin

The social norms and values of the patients’ families of origin are described as forming social expectations from the family, also affecting the patients. The mentioned expectations are often related to education and career but attempts of trying to fit in are also represented.

“The pressure that I have to perform comes from the family. They are high achievers in the family with good jobs.”

There are examples of family values and norms perceived as being overprotective or lacking in expectations, which are also perceived as contributing to the problem. One patient describes being torn between the norms and values at home and those of the surroundings during childhood. Values and norms of the family of origin related to talking about problems and distress are also represented as well as burdensome expectations from others on starting a family life.

“He has grown up with the idea that you shouldn’t talk about your mental problems. He is unsure if it is something that comes from his culture or from society in general.”

#### Future social objectives

This sub-theme illuminates the patients’ hopes and goals for the future, how they want to feel, function and be able to perform further on in life. Visions of a desired social position are also represented.

“Want to have normal standards like when I grew up.”

Hopes of finding a meaning and worries of what will happen if they do not get better are also represented.

### Making sense of the problem

This theme covers the patients’ perceptions of causes of, or contributing factors to, their current distress. They relate their problems to recent external stressful conditions, often work related, or to difficult family and childhood-related conditions from the past. Other perceived causes are internalized, e.g. hereditary or biological, and suspicions of a psychiatric diagnosis or psychological vulnerability are also represented. The sub-themes are: Family and childhood related conditions, External stressful conditions, Heredity and biology, Psychological vulnerability, and Suspicion of a psychiatric diagnosis.

#### Family- and childhood-related conditions

The most frequently mentioned perceived causes of, or contributing factors to, the distress are related to difficult family- and childhood-related conditions. These are often conditions of substance abusing parents or other family members.

“She thinks that her upbringing with an alcoholic father and a single mother, who later died due to illness, has a big impact on how she feels today.”

Some patients mention loss of family members, often during childhood, as traumatic experiences still affecting them. There are also descriptions of an upbringing without limits and boundaries, absent or spoiling parents, perceived as contributing factors. Other examples are too demanding conditions during upbringing, contributing to current low self-confidence. A difficult situation in school with experiences of exclusion and bullying is also frequently reported as contributing to the perceived distress.

“I was good at school but teased. Didn’t have a friend. I was alone and felt worthless…. It has greatly affected my self-esteem. I easily feel left out.”

#### External stressful conditions

Other frequently reported factors perceived as contributing to current distress are recent external stressful conditions. These are often related to a stressful working situation or being out of job. Sometimes the job-related stress is caused by exclusion or bullying at work. The external stressful conditions are often described as contributing or triggering but are not perceived as the actual cause of the problem.

“A lot of work was the trigger but not the real cause.”

Some patients describe the stressful conditions as worsening the symptoms of distress. These are often of relational character, like experiences of separation, infidelity or of feeling responsible for sick family members.

“He explains his condition by saying that some difficult things have happened in his life. The divorce two years ago.”

Sometimes the cause of the problems is perceived to be a combination of a stressful job situation, difficult experiences from the past and relational problems. There are also examples of changes of context and a high-performance environment contributing to the perceived distress.

#### Heredity and biology

Commonly described causes of the perceived distress are heredity or biological factors. The patients describe how they recognize their experienced distress or symptoms among family members.

“Biological factors. My mother has also had a mental illness, a lot of anxiety.”

The patients reflect on genetics and how their genes contribute to their current distress, and are curious about neurological contributing factors.

#### One’s own psychological vulnerability

Some contributing factors were described by the patients as having more of a personality or psychological character, in this sub-theme captured as a psychological vulnerability. There are descriptions of patients’ perceived dysfunctional personality traits, making it difficult to perform in accordance with norms and social values. These are personality traits making the patients feel strange or that they don’t fit in in their social context.

“I can’t put my finger on it, and neither can others. It’s more of a personality trait.”

Other personality traits perceived as troublesome in relation to norms and social expectations are controlling tendencies as well as being lazy and sloppy. Personality traits of a more emotional character like being negative or too emotional are also described as problematic in relation to norms and social acceptance.

“It could be because of certain personality traits, I see the negative in things, situations. I see what can go wrong, put the emphasis on the difficult, negative.”

The psychological vulnerability is also related to the way the patients relate or react to external factors, and sometimes this vulnerability results in an inability to adapt to changes. Sometimes the patients’ vulnerability is related to their interpretation of things that happen to them, and their abilities.

“I have had a superb family and friends. Actually, I have no problem if you look at it objectively. But I overanalyse, am negative.”

Some patients describe a neglect of self-care or one’s own needs.

#### Suspicion of a psychiatric disorder

Some patients describe the causes of their current distress in terms of psychiatric diagnoses, referring to some disorders. The psychiatric diagnoses mentioned are used to describe the problem, but also contain explanations of the perceived distress. Some patients feel more certain, and some describe their suspicions of a psychiatric diagnosis.

“Don’t know if it’s that I have recurrent depression.”

Also represented are reflections of a psychiatric diagnosis being a root cause, linked to other contributing stress factors.

### Experiences of, and wishes for, help

This theme contains information about previous help-seeking experiences, including non-medical help seeking, and perceived barriers to help seeking. Information is also found about what the patients believe would be helpful as well as possible concerns related to healthcare interventions. The sub-themes are: Ideas and thoughts about improvement, Help-seeking experiences, Non-medical help seeking, Barriers to help seeking, and Concerns related to healthcare interventions.

#### Help-seeking experiences

Here previous help-seeking experiences and treatment are illustrated. The described experiences of healthcare interventions are recent as well as from the past and there are sometimes many.

“Have tried different medicines and therapies. I’m standing on this spot. Wondering if there is anything else to try.”

The patients describe healthcare interventions perceived as helpful from the past, including medication, counselling and psychological treatment. Disappointments related to healthcare interventions are frequently described, as well as interventions that have not been helpful. The disappointments are related to a feeling of not getting better but also changes of doctors or lack of follow up. Feelings of being given the run around as well as a long wait for an appointment are also represented, as are examples of “not being ill enough” to receive care and treatment.

“They asked if I wanted to hurt myself and when I said no, they said unfortunately they couldn’t help me.”

#### Non-medical help seeking

The patients share their experiences of non-medical help seeking, for example, talking to friends and seeking information on the Internet. Physical activities like dancing and practicing yoga are mentioned as well as mindfulness and acupuncture. There are also examples of support from non-medical professionals.

“I have talked to friends, read a lot online and practiced some yoga. Previously I danced quite a lot of salsa.”

Seeking help through spiritual- or church-related activities are also described by some patients. Other examples of non-medical help seeking are reading books, taking vitamins, and using drugs.

“The drugs have helped me deal with my anxiety and worry.”

#### Barriers to help seeking

In the sub-theme Barriers to help seeking, the patients describe their reasons for not seeking help. Some patients express how they did not want to seek help, sometimes because of a desire to manage on their own and sometimes because of bad experiences from the past. Some patients did not seek help because of shame or a denial of the problem, or because of a fear of not being taken seriously.

“Feelings of shame. I don’t feel bad. I don’t seek help. I’m strong. Others seek help.”

Economic reasons are mentioned as a barrier to help seeking as well as the long wait for an appointment. Some patients experience a lack of information preventing them to seek help and ignorance of what help they need. There are also examples of doubt in the efficiency of healthcare interventions and descriptions of difficulties in seeking help when being ill.

“It can be difficult that you, in contact with healthcare, have to be so driven yourself. It is difficult to actively seek help when you are depressed.”

#### Ideas and thoughts about improvement

In this sub-theme the patients give examples of what they believe would be helpful in their present situation. Frequently expressed are medication and counselling or psychological treatment. Another healthcare intervention often mentioned and desired is a diagnostic assessment, often expressed as a way of understanding the problems better which could be used as a foundation for further treatment.

“I wanted to come here to find out if I have ADHD or if it’s something else.”

A desire to obtain coping strategies for managing their distress better or for changing their way of thinking is also frequently described. Some patients describe a need for help with managing their everyday life. Thoughts about activities perceived to be helpful are also expressed by some patients, as steps towards feeling better.

“The best thing for me is to write, to be creative.”

Some patients express that they don’t know what would be helpful or how they do not need any help.

#### Concerns related to healthcare interventions

Here the patients’ concerns related to healthcare interventions are presented. An example of these concerns is a fear of not being taken seriously or not receiving any help. Examples of a lack of trust towards healthcare professionals and a fear of not obtaining adequate treatment are also expressed.

“Afraid of ending up on the wrong path again, that the doctor won’t give me the right medicine. Difficult to trust in healthcare, having been misjudged and mistreated so many times.”

## Discussion

### Main findings

The study aimed to find out what information the CFI questions revealed when used with native Swedish-speaking patients, as a part of routine clinical psychiatric assessment at outpatient settings in Stockholm. An additional aim was to enhance understanding of what kind of information the questions about background and identity yielded when used with these patients. From a thematic analysis of the CFI answers the following themes were identified:

Descriptions of distress and dysfunction, Managing problems and distress, Current life conditions affecting the person, Perceived failure in meeting social expectations, Making sense of the problem, and Experiences of, and wishes for, help.

The most comprehensive theme, named Descriptions of distress and dysfunction, comprises information about how the patients described their distress and its limiting consequences for social relations and functioning in everyday life. Patients used emotional expressions in their descriptions and behaviours related to dysfunction, and the use of psychiatric terms was common and included in their everyday language. The psychiatric terms were used to describe the problems but at the same time revealed perceived causes of distress. These results differ from the ones of a previous study, from an analysis of the CFI answers from non-native speaking patients in the same setting, where the use of psychiatric terms was not common and where emotional expressions were often tightly linked to somatic ones (18). The problems were also presented from a personal historical perspective, describing when they first emerged.

The patients described how they currently managed their problems and distress in everyday life, sometimes through strategies, such as activities and routines. They shared their current self-positioning to their problems, which was sometimes through active neglect and sometimes through feelings of shame. There was also information concerning whether the patients told others about their problems, with whom they shared and how they talked about them.

The CFI answers provided a thick description of current life conditions, including social stress factors related to work and/or economy. The answers also gave rich information about their own and others’ explanations of the problems and what would be helpful. Through the CFI, the patients shared information about how they made sense of the problem, and their perceptions of causes, or contributing factors, to their current distress. The patients’ illness explanations varied widely from current external stressful conditions, or difficult family and childhood related conditions from the past to hereditary or biological factors. Several patients also shared suspicions of a psychiatric disorder. Furthermore, through the CFI the clinician obtained information about the patient’s previous help-seeking experiences and what was helpful or not helpful, and what the patients believed would be helpful for them now.

The information about the patients’ approach and strategies to manage the problems, as well as information about sharing or not sharing the problems with others, correspond with the thematic analysis of the CFI answers from non-native Swedish speaking patients from the same setting (18). This was also partly the case regarding information gained on social determinants of health and the patients’ views of what is causing the problem. The CFI has been criticized for not eliciting social structures and determinants of health sufficiently (19). However, previous research found the CFI to contribute with information about several aspects of social structures and determinants of mental health problems, such as housing and financial instability and poor access to healthcare (25), corresponding with our results.

### Findings on identity and perceived failure in social expectations

We wanted to understand better what information the CFI questions about background and identity gave when used with native Swedish-speaking patients. Previous research has identified the CFI questions about background and identity as abstract and difficult for some patients to understand (6, 7, 14, 20, 21). For patients in this study, the CFI questions about background and identity mainly facilitated sharing information about their social position and identification with a social role. Identity was often discussed in relation to sociocultural expectations and the ability to perform accordingly. These expectations were described as formed by their current sociocultural context and sometimes by the culture of the family of origin. Expectations were often related to education and career, but there were also examples of attempts of trying to “fit in” into a social group. The patients valued their self-worth in relation to their ability to perform in accordance with norms and values in their context. Not being able to perform based on perceived expectations, one’s own or others’, led to feelings of failure and stress. Feelings of belonging to a certain social or cultural group, like working class, was found among some patients, and feelings of losing ones’ identity, of not belonging or feelings of being lost. Desires of a sense of belonging to a certain social or cultural group or to be like “everyone else” were also expressed.

How patients talked about cultural identity and of belonging to a social or cultural group in this study differs from non-native Swedish-speaking patients in the same settings (18). For the non-native speaking patients, the CFI questions about background and identity stimulated sharing of information about a sense of belonging related to the culture of origin in relation to the Swedish culture. The patients expressed feelings of being split between their culture of origin and the Swedish culture, and of no longer belonging to either. (18). Another study on minority patients found the CFI identity questions to stimulate sharing of information about the impact of migration and changes of cultural contexts (11). These questions have also been found to reveal illness identities and clinical symptoms related to social, economic, and political structures when used with minority patients (16).

In our studies, the CFI questions on cultural identity seem to be interpreted and understood in different ways by native speaking patients (cultural majority patients) and non-native speaking patients (migrants/cultural minority patients). Depending on how the questions are understood, they may elicit different kinds of information. If the questions are interpreted primarily in terms of social position, there is a risk that other aspects of cultural identity relevant to health are not revealed. It is possible that a less abstract way of obtaining information about a patient’s cultural identity, and its perceived impact on health, could be to ask about a sense of belonging (18).

The patients in this study perceived that the high expectations on oneself of social roles to manage and fulfil often result in poor health and distress. Feelings of social failure and of alienation from sociocultural norms were described. The high expectations of fulfilment of social roles and related feelings of social failure could possibly be related to culture and values in Swedish society. Results from reoccurring World Values surveys show that Sweden is unique in its extremely secular and individualistic values (26). Swedish society is strongly marked by valuing the meritocratic system, a system where appointments and responsibilities are assigned to individuals based on their merits (27). These merits can be determined through evaluations or examinations but can also be earned by intellectual or manual labour, as each person has his or her own talents. In a meritocratic system, individuals can, theoretically, reach any goal (28). In the Swedish social environment of individualism and self-expression values, where education including higher education is free of charge, the individual is to a high degree responsible for their own success, and no one else is to blame when not fulfilling one’s own expectations.

Many patients in this study express feelings of being worthless, which could be interpreted in different ways. It is possible that these expressions are mainly signs of depression, on the other hand, the low self-worth could also be related to sociocultural expectations and norms. Shared or not shared cultural values between the patient and the clinician, related to social expectations and norms, may affect clinicians’ interpretation of patients’ presentation of distress, low self-worth, and of social failure. Concerning the use of psychiatric concepts, the philosopher Fredrik Svenaeus (29) notes how cultural norms and values might affect the perceived function of psychiatric diagnostic labels. Cultural values of great confidence in medical explanations, for example, sometimes offer comfort in diagnostic labels, providing a possible identity and affiliation that might function as an explanation for social failure or suffering. It is possible that the high frequency of suspicions of neuropsychiatric disorders expressed by the patients in this study partly represents a medicalisation of a feeling of social failure. Cultural and social norms and values of majority culture individuals are often not consciously recognized or reflected upon, by individuals or in Swedish healthcare contexts, (30–32), which can make the direct CFI questions about cultural identity difficult for healthcare workers to understand. Culture and norms may be taken for granted or inaccurate assumptions are made about uniformity of cultural or social experiences. However, when such reflection is permitted to take place, it may provide a new awareness and possibility for valuable information to emerge of relevance for understanding the patient’s perception of the problem and for treatment planning.

Even though the perceived expectations were sometimes described as shaped by their family of origin, these native Swedish-speaking patients usually did not relate cultural identity to ethnicity or background, but more to socioeconomic norms in their present context and expectations of a social position. Therefore, combining questions about cultural identity and background may not facilitate exploration of cultural and contextual factors of importance for the current health situation. The difficulties associated with asking about identity together with background, have been identified in focus group interviews with clinicians in previous research (7). However, the CFI questions about identity stimulated sharing of information about the patient’s own norms, values, and views of their social failure, pointing to the importance of support in finding a social role, and treatment which is not only symptom reducing but also focused on self-respect or acceptance.

### Trustworthiness and rigour

The patients selected for this analysis had Swedish as their native language, but we do not know if some patients belong to any ethnic minority group, or have an ethnicity (e.g. from one parent) other than Swedish. The analysis of our data was not done using the actual CFI with the patients but with the clinicians’ documentation of the patients’ CFI answers. The interviews were often documented verbatim. However, we do not know what was actually said during the interviews which limits the credibility of our results (33).

The large dataset, containing rich and nuanced information from 62 patients’ answers to the CFI questions, documented in detail by several, CFI-trained clinicians, supports the credibility of the study (33), through the collection and thorough analysis of patients’ perspectives resulting in unique information presented about integrating cultural, social and contextual factors of importance for the patients. We had enough data to cover significant variations and the comprehensive material enabled us to quantify the frequency of quotes for each theme, providing an added value to the qualitative analysis. Although the frequency of references is not the purpose of qualitative analysis, certain patterns of quantizing responses provide clues of relevance of the presented results (34).

The thorough analysis process involving three researchers overseeing the meaning units, sub-themes and themes several times until consensus was reached, as well as using quotes exemplifying our analysis, was important and a way to address rigour and dependability (33).

### Conclusion and clinical implications

When using the CFI with native Swedish-speaking patients, functioning as a proxy for cultural majority patients, in a suburban setting in Stockholm, a thick description of how the patients describe, manage, and make sense of their problems, as well as contextual information including current social factors, was given. The CFI questions about cultural identity gave rich information about the patient’s own perception related to their social position and feelings of social defeat. For further adaptation and application of the CFI, we see a need for re-framing the questions about cultural identity and its impact on health so that they are better understood by cultural majority population patients. This is suggested as direct questions about culture may be difficult to understand when cultural norms are implicit and not reflected upon. For clinical implications, our findings suggest that for cultural majority patients the DSM-5 CFI can be a useful person-centred tool for exploring cultural and, in particular, social factors and patients’ perceptions and understandings of distress.

## Data availability statement

The datasets presented in this article are not readily available because of confidentiality reasons. Requests to access the datasets should be directed to Sofie Bäärnhielm, sofie.baarnhielm@regionstockholm.se.

## Ethics statement

The studies involving humans were approved by Regional Ethical Review Board in Stockholm (2015/243-31/2). The studies were conducted in accordance with the local legislation and institutional requirements. The participants provided their oral informed consent to participate in this study.

## Author contributions

MW: Conceptualization, Data curation, Formal analysis, Methodology, Software, Writing – original draft, Writing – review & editing. VD: Conceptualization, Validation, Writing – review & editing. LN: Conceptualization, Validation, Writing – review & editing. SB: Conceptualization, Formal analysis, Funding acquisition, Supervision, Validation, Writing – review & editing.

## Footnotes

1 Due to birth or upbringing.
